# miR-20a regulates proliferation, differentiation and apoptosis in P19 cell model of cardiac differentiation by targeting Smoothened

**DOI:** 10.1242/bio.019182

**Published:** 2016-08-19

**Authors:** Feng Ai, Yanwei Zhang, Bangtian Peng

**Affiliations:** Department of Cardiovascular Surgery, Henan Provincial People's Hospital and People's Hospital of Zhengzhou University, Zhengzhou 450000, People's republic of China

**Keywords:** miR-20a, P19 cell, Proliferation, Differentiation, Apoptosis, Smoothened

## Abstract

MicroRNA (miR)-20a, a member of the miR-17-92 cluster related to cardiac development, was obviously downregulated in myocardially differentiated P19 cells compared with normal P19 cells. Smoothened (SMO) is a member of the Hh pathway. Hh signaling induces cardiac differentiation in P19 cells, and SMO mediates the Hh pathway during embryonic development. Using bioinformatic prediction software Targetscan (http://www.targetscan.org/), PicTar (http://pictar.bio.nyu.edu), and miRBase (http://microrna.sanger.ac.uk/), miR-20a and the 3′-untranslated region (3′-UTR) of SMO mRNA were predicted to have complementary binding regions. Accordingly, we inferred that miR-20a might act as a regulator of SMO, and regulate proliferation, differentiation and apoptosis in P19 cells. We determined the expression of miR-20a, SMO and marker proteins of cardiomyocytes (cTnT, GATA4 and desmin) by quantitative real-time PCR (qRT-PCR) and western blot assays, and found that P19 cells had differentiated into cardiomyocytes successfully at differentiation day 10, and downregulation of miR-20a and upregulation of SMO existed in myocardially differentiated P19 cells. Cell proliferation, differentiation and apoptosis detection showed that miR-20a upregulation inhibited proliferation and differentiation and enhanced apoptosis in P19 cells. Moreover, we verified that miR-20a directly targeted SMO and knockdown of SMO and miR-20a overexpression had similar effects on P19 cell proliferation, differentiation and apoptosis, which verified the speculation that miR-20a inhibits proliferation and differentiation and enhances apoptosis in P19 cells by directly targeting SMO. Our results suggest that miR-20a may be a potential target against congenital heart diseases.

## Abstract (precis)

**Summary:** Our findings may bring a new viewpoint into the mechanisms of cardiac abnormalities and suggest that miR-20a may be a potential new therapeutic target for congenital heart diseases.

## INTRODUCTION

The vertebrate heart, derived from the mesodermal cells, is the first functional organ forming in vertebrate development (Zaffran and Frasch, 2002). The formation of a mature normal heart is a complicated course which depends on the sequential expression of multiple genes and various pathways, including the Hedgehog (Hh), the BMP, the Notch, and the Wnt/β-Catenin pathway (Bajolle et al., 2009; Miyazono et al., 2010). Many studies have reported that any deletions or mutations in the above procedures are likely to cause cardiac malformation (Qin et al., 2013; Zhao et al., 2007; Zhu et al., 2015). Congenital heart defect (CHD) is the most common disease of the newborn and accounts for about 40% of perinatal deaths and over 25% of deaths within the first month of life (Trojnarska et al., 2009). The impaired process of embryonic stem cell differentiation into cardiomyocytes caused by gene deletions or mutations is the primary cause of CHD (Hoffman, 1995; Hoffman and Kaplan, 2002), thus, better understanding of the molecular mechanism of embryonic stem cell differentiation into cardiomyocytes will contribute to finding effective approaches for CHD treatment.

MicroRNAs (miRNAs, miRs) are a class of single-stranded, non-coding, small RNA molecules (19∼23 ribonucleotides) which regulate gene expression by targeting the 3′-untranslated region (3′-UTR) of mRNAs for translational repression and/or degradation (Wienholds and Plasterk, 2005). miRs are recently found to be activated in CHDs (Kehler et al., 2015; Van Rooij and Olson, 2007; Xu et al., 2009), and many studies have confirmed a relationship between miRs and cardiogenesis (Catalucci et al., 2008; Thum et al., 2008).

P19 cells are pluripotent stem cells and have an ability to differentiate into multiple cell types. Low concentrations of dimethyl sulfoxide induce P19 cells to differentiate into cardiomyocytes. Therefore, P19 cells are used widely as a myocardial cell model in myocardial differentiation to elucidate the molecular mechanism that controls their proliferation and differentiation, which may give a new insight into the underlying mechanisms of heart development.

Matured miR-20a-5p as a member of the miR-17-92 cluster associated with cardiac development was obviously downregulated in cardiomyocytes compared with normal P19 cells (Hu et al., 2011); however, how miR-20a plays a role in the differentiation of P19 cells into cardiomyocytes is unknown. The Hh pathway is critical in the process of embryonic development. Gianakopoulos et al. found that the Hh pathway induces cardiomyogenesis in P19 cells (Gianakopoulos and Skerjanc, 2005). Smoothened (SMO), a member of the Hh pathway, mediates Hh pathway during embryonic development (Yang et al., 2010) and with the application of bioinformatic software Targetscan (http://www.targetscan.org/), PicTar (http://pictar.bio.nyu.edu), and miRBase (http://microrna.sanger.ac.uk/) miR-20a and SMO mRNA 3′-UTR were found to have possible binding sites. Therefore we deduced that miR-20a might reversely regulate the expression of SMO by binding to the 3′-UTR of SMO mRNA, thereby regulating proliferation, differentiation and apoptosis in P19 cells. In the present study, we aimed to display the role and regulatory mechanism of miR-20a in heart development, which will also provide new insights into finding novel and effective therapeutic approaches for CHD treatment.

## RESULTS

### miR-20a is underexpressed and SMO is overexpressed in P19 cells at differentiation day 10

**Fig. 1.**
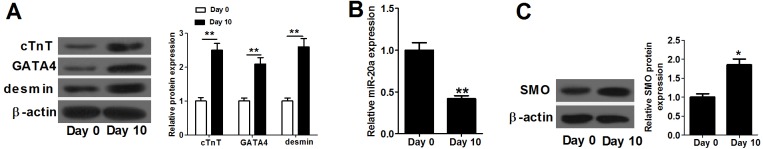
**miR-20a is downregulated and SMO is upregulated in myocardially differentiated P19 cells.** (A) Western blot assay shows that the expression levels of marker proteins of cardiomyocytes cTnT, GATA4 and desmin were obviously higher in P19 cells at differentiation day 10 than those in P19 cells at differentiation day 0. (B) qRT-PCR shows that compared with P19 cells at differentiation day 0, P19 cells at differentiation day 10 have an obvious decrease in miR-20a expression. (C) The expression level of SMO protein is significantly higher in P19 cells at differentiation day 10 than that in P19 cells at differentiation day 0. Error bars indicate s.d., *n*=3. *P<0.05 and **P<0.01 (Student's *t*-test).

To assess whether P19 cells showed cardiomyocyte differentiation, P19 cells were harvested at day 0 and 10 of differentiation, and western blot assay was conducted to detect the levels of cTnT, GATA4 and desmin. The expression levels of cTnT, GATA4 and desmin were obviously higher in P19 cells at differentiation day 10 than those in P19 cells at differentiation day 0, which confirmed that P19 cells had differentiated into cardiomyocytes successfully at differentiation day 10 (Fig. 1A). Then we measured the expression levels of miR-20a and SMO in P19 cells at day 0 and 10 of differentiation by qRT-PCR and western blot analysis. Compared to P19 cells at differentiation day 0, P19 cells at differentiation day 10 showed significant downregulation of miR-20a and obvious upregulation of SMO protein (Fig. 1B and C).

### miR-20a overexpression inhibits proliferation and differentiation and accelerates apoptosis in P19 cells

**Fig. 2.**
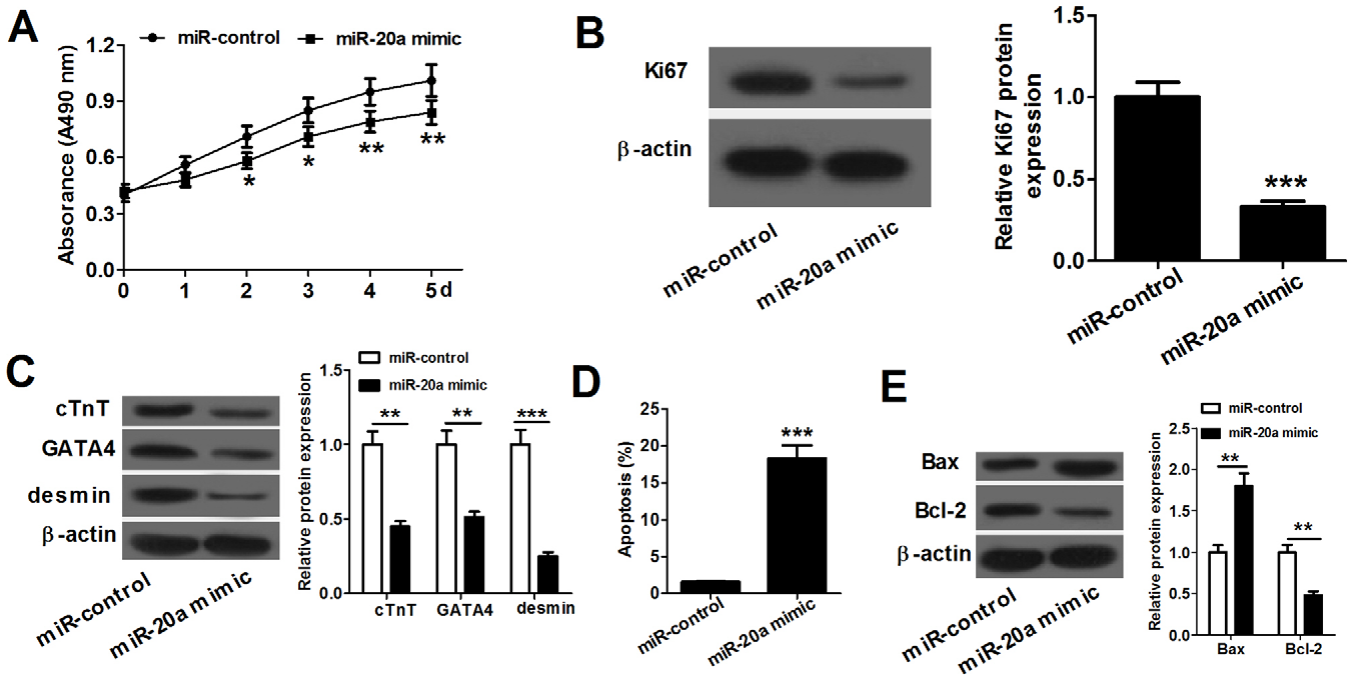
**miR-20a overexpression suppresses proliferation and differentiation and promotes apoptosis in P19 cells.** (A) P19 cells transfected with miR-20a mimics proliferate significantly slower than those transfected with miR-control. Error bars indicate s.e.m. (*n*=3). (B) The expression level of Ki67 protein is obviously decreased in miR-20a-overexpressing P19 cells compared with that in control cells. Error bars indicate s.d. (*n*=3). (C) P19 cells transfected with miR-20a mimics have obvious decreases in cTnT, GATA4 and desmin protein expression compared with the control cells. Error bars indicate s.d. (*n*=3). (D) P19 cells transfected with miR-20a mimic have significantly higher apoptosis rate than the control cells. Error bars indicate s.d. (*n*=3). (E) P19 cells transfected with miR-20a mimics have a marked increase in Bax protein expression and an obvious decrease in Bcl-2 protein expression compared with the control cells. Error bars indicate s.d., *n*=3. *P<0.05, **P<0.01 and ***P<0.001 (Student’s *t*-test).

To investigate the role of miR-20a in P19 cells we transfected P19 cells with miR-20a mimics and miR-control, and performed proliferation, differentiation and apoptosis detection. Cell proliferation assay showed that P19 cells transfected with miR-20a mimics proliferated significantly slower than those transfected with miR-control (Fig. 2A). The expression levels of proliferation-related protein Ki67 were reduced in miR-20a-overexpressing P19 cells compared with those in control cells (Fig. 2B). The effect of miR-20a on the differentiation of P19 cells into cardiomyocytes was investigated by measuring the levels of three marker proteins of cardiomyocytes cTnT, GATA4 and desmin. As shown in Fig. 2C, miR-20a overexpression decreased the cTnT, GATA4 and desmin protein expression in P19 cells compared with the control cells, which showed that miR-20a represses the differentiation of P19 cells into cardiomyocytes. The impact of miR-20a overexpression on P19 cell apoptotic response was assessed by flow cytometry analysis. As shown in Fig. 2D, the overexpression of miR-20a obviously increased the apoptosis rate of P19 cells. In addition, miR-20a overexpression increased Bax protein expression and decreased Bcl-2 protein expression in P19 cells compared with the control cells, indicating that miR-20a promotes P19 cell apoptosis (Fig. 2E). Altogether, the data manifested that miR-20a inhibits proliferation and differentiation and accelerates apoptosis in P19 cells.

### SMO is a target of miR-20a

**Fig. 3.**
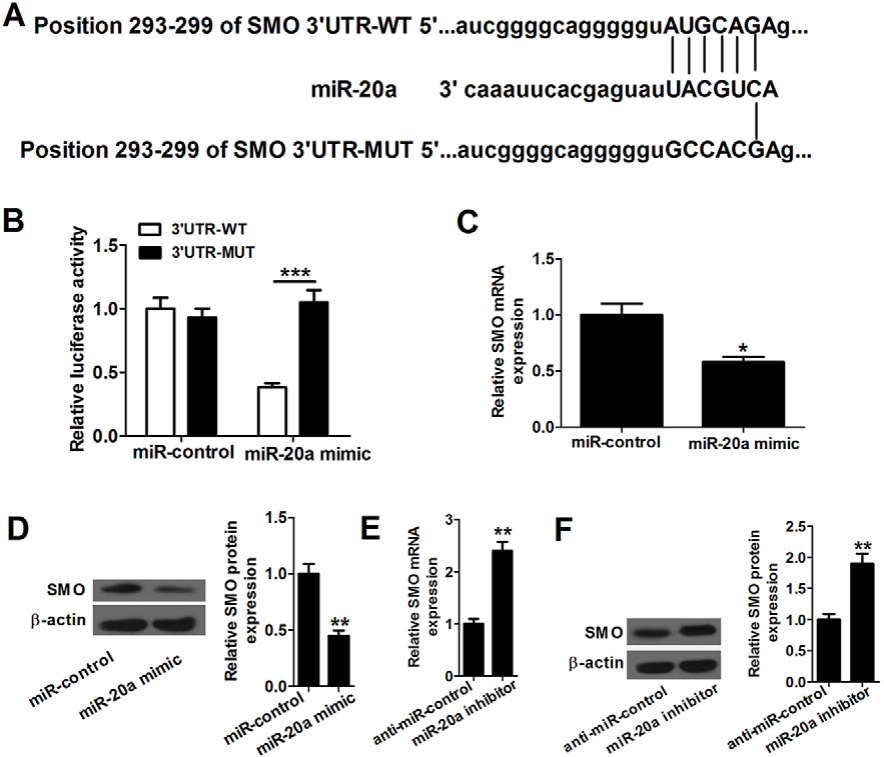
**SMO is a direct target of miR-20a.** (A) Bioinformatics-based target prediction analysis shows that SMO is a potential target of miR-20a and the binding site is on the 3′-UTR of SMO. Black lines indicate the predicted binding sites. (B) P19 cells co-transfected with plasmid containing 3′-UTR-WT regions of SMO and miR-20a mimics have significantly decreased luciferase activity compared with control, while mutation of the putative miR-20a binding sites in the 3′-UTR regions of SMO abolishes this effect. (C,D) Overexpression of miR-20a inhibits SMO mRNA and protein expression in P19 cells. (E,F) Downregulation of miR-20a enhances SMO mRNA and protein expression in P19 cells. *P<0.05, Error bars indicate s.d., *n*=3. **P<0.01 and ***P<0.001 (Student’s *t*-test).

To study the molecular mechanisms by which miR-20a would suppress proliferation and differentiation and promote apoptosis in P19 cells, we searched for miR-20a targets with online gene analysis software TargetScan (http://www.targetscan.org/), PicTar (http://pictar.bio.nyu.edu/) and miRBase (http://microrna.sanger.ac.uk/), and found that SMO was a predicted target of miR-20a (Fig. 3A). We also found that P19 cells co-transfected with plasmids carrying wild-type 3′-UTR SMO and miR-20a mimics had less luciferase activities than control, but mutation of the putative miR-20a binding sites in the 3′-UTR regions of SMO abolished this effect (Fig. 3B). qRT-PCR and western blot assays showed that overexpression of miR-20a suppressed SMO mRNA and protein expression, and down-expression of miR-20a increased SMO mRNA and protein expression in P19 cells which strongly validated a post-transcriptional regulation of SMO protein by miR-20a (Fig. 3C-F). Altogether, these results suggested that miR-20a directly targets SMO in P19 cells.

### miR-20a regulates P19 cell proliferation, differentiation and apoptosis by targeting SMO

**Fig. 4.**
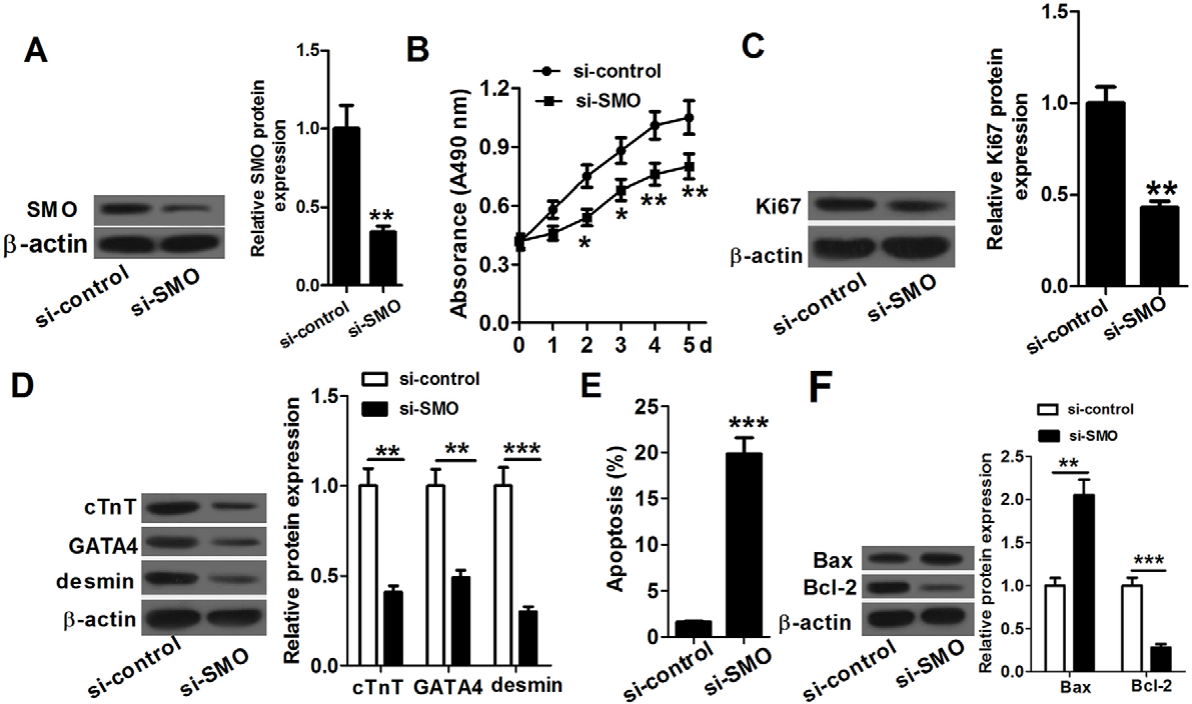
**Knockdown of SMO suppresses proliferation and differentiation and promotes apoptosis in P19 cells.** (A) P19 cells transfected with si-SMO had lower SMO protein expression than control cells. Error bars indicate s.d. (*n*=3). (B) P19 cells transfected with si-SMO proliferate significantly slower than those transfected with si-control. Error bars indicate s.e.m. (*n*=3). (C) The expression level of Ki67 in P19 cells transfected with si-SMO is obviously lower than that in control. Error bars indicate s.d. (*n*=3). (D) P19 cells transfected with si-SMO have obvious decreases in cTnT, GATA4 and desmin protein expression compared with the control cells. Error bars indicate s.d. (*n*=3). (E) Knockdown of SMO results in an obviously increased apoptosis in P19 cells. Error bars indicate s.d. (*n*=3). (F) P19 cells transfected with si-SMO have a marked increase in Bax expression and an obvious decrease in Bcl-2 expression compared with the control cells. Error bars indicate s.d., *n*=3. *P<0.05, **P<0.01 and ***P<0.001 (Student’s *t*-test).

If miR-20a suppressed proliferation and differentiation and promoted apoptosis in P19 cells which were indeed regulated by SMO, we would expect that knockdown of SMO would have similar effect. To test this hypothesis, we transfected P19 cells with si-SMO or si-control, and then detected cell proliferation, differentiation and apoptosis. Western blot assay showed that P19 cells transfected with si-SMO had lower SMO expression than control cells. Results of the cell proliferation assays showed that P19 cells transfected with si-SMO proliferated significantly slower than those transfected with si-control (Fig. 4B). The levels of Ki67 in P19 cells transfected with si-SMO were obviously lower than that in control (Fig. 4C). Results of cell differentiation detection showed that P19 cells transfected with si-SMO had an obvious decrease in cTnT, GATA4 and desmin expression compared to the control group (Fig. 4D). Results of cell apoptosis analysis revealed that knockdown of SMO resulted in an obviously increased apoptosis in P19 cells (Fig. 4E). Furthermore, P19 cells transfected with si-SMO had an increase in Bax expression and a decrease in Bcl-2 expression compared to the control cells (Fig. 4F). These data showed that knockdown of SMO suppresses proliferation and differentiation and accelerates apoptosis in P19 cells, which confirmed our inference that si-SMO could mimic the role of miR-20a. Collectively, these results manifested that miR-20a suppresses proliferation and differentiation and accelerates apoptosis in P19 cells by directly targeting SMO.

## DISCUSSION

CHD, which includes multiple structural and functional abnormalities of the heart and great vessels, is the most common type of birth defects and also the leading cause of infant morbidity and mortality (Capozzi et al., 2008; Eleftheriades et al., 2012). miRNAs, widely distributed in animals and land plants, post-transcriptionally modulate gene expression and participate in various biological processes, including development, cell survival, proliferation, and differentiation (Axtell and Bowman, 2008; Bartel, 2004). Computational predictions indicate that 60% of protein-encoding genes are modulated by miRNAs in humans (Friedman et al., 2009). Of these miRNAs, many have been found to be associated to cardiogenesis and act as new biomarkers and potential targets for CHD. For instance, miR-19b overexpression promotes proliferation and differentiation but suppresses apoptosis in P19 cells by inactivating the Wnt/β-catenin pathway, and miR-20b overexpression enhances apoptosis and promotes differentiation in P19 cells through activating the BMP pathway (Qin, 2013; Zhu, 2015). These findings suggest that miR-19b and miR-20b may represent potential targets for CHD treatment and provide novel insights into the molecular mechanisms underlying heart diseases. In order to find new therapeutic targets for CHD treatment and obtain a better understanding of the mechanisms underlying cardiac diseases, more studies are needed to assess the roles of miRNAs in cardiogenesis.

Some reports have showed that the miR-17-92 cluster, containing 6 mature miRNAs (miR-17, miR-18a, miR-19a, miR-19b-1, miR-20a, and miR-92-1), plays important roles in the development of lung, heart, blood and vessel. For example, (Lu et al., 2007) reported that upregulation of miR-17-92 enhances proliferation and suppresses differentiation of lung epithelial progenitor cells. Ventura et al. documented that the mice lacking miR-17-92 have severely ventricular septal defects in the hearts, thereby leading to smaller embryos and postnatal death, which suggests an important function of this cluster during cardiac development (Ventura et al., 2008). Moreover, miR-18 and miR-19 modulate CTGF and TSP-1 expression and the two proteins are important to healthy cardiac ageing, which suggests that these miRNAs are involved in age-related cardiac remodelling (van Almen et al., 2011). miR-20a, as a member of the miR-17-92 cluster, was reported to have been dysregulated in heart failure patients treated with cardiac resynchronization therapy and inhibit stress-induced cardiomyocyte apoptosis by targeting Egln3/PHD3 (Marfella et al., 2013; Frank et al., 2012). Recently, miR-20a is obviously downregulated in myocardially differentiated P19 cells compared with normal P19 cells and may be functionally related to cardiogenic processes, though its precise role remains elusive.

In the present study, we used P19 cells as a myocardial cell model to establish a miR-20a overexpression cell line and investigated the roles of miR-20a in cardiac differentiation. We found that miR-20a upregulation suppresses proliferation and differentiation and promotes apoptosis in P19 cells, and studied the molecular mechanisms by which miR-20a overexpression has the same effects. With the application of bioinformatic predictions, we identified several potential binding targets of miR-20a, and chose SMO for further study. SMO is a transmembrane protein that can activate the downstream Hh pathway. Several recent studies have documented that some small synthetic inhibitors suppress the Hh signalling pathway by specifically binding to SMO or antagonizing the activity of SMO (Chen et al., 2002a,b; Frank-Kamenetsky et al., 2002). The Hh signalling pathway is a primary embryonic signalling cascade that modulates stem cell and progenitor cell differentiation in a great many developmental processes, and can induce cardiomyogenesis in P19 cells (Gianakopoulos and Skerjanc, 2005; Ingham and Placzek, 2006). Dual-luciferase assay verified that miR-20a targets SMO directly and the expression levels of SMO mRNA and protein were modulated by miR-20a. Although some targets of miRNA-20a are enriched in cell cycle regulating genes, knockdown of SMO suppressed proliferation and differentiation and enhanced apoptosis in P19 cells, which could mimic the effect of miR-20a overexpression. All the data suggested that miR-20a inhibits proliferation and differentiation and promotes apoptosis in P19 cells at least partly by directly targeting SMO.

In summary, downregulation of miR-20a and upregulation of SMO exist in myocardially differentiated P19 cells. We provided evidence to verify that SMO is a binding target of miR-20a, and miR-20a modulates proliferation, differentiation and apoptosis of P19 cells by targeting SMO. These data may provide novel insights into the regulatory mechanisms underlying heart diseases. Moreover, miR-20a may be a possible target for the treatment of CHD. Consequently, the next step is to study whether dysregulation of miR-20a contributes to CHD *in vivo*.

## MATERIALS AND METHODS

### Cell culture and induction of differentiation

P19 cells were obtained from the American type culture collection (ATCC; Manassas, VA, USA). Cells were cultured in α-minimal essential medium (α-MEM; Invitrogen, Melbourne, VIC, Australia) supplemented with 10% fetal bovine serum (FBS; Gibco BRL, Grand Island, NY, USA), 100 µg/ml streptomycin, and 100 U/ml penicillin in a humidified incubator containing 5% CO_2_ at 37°C. To induce cardiac differentiation, cells were cultivated as aggregates for 4 days in 1% α-MEM containing 10% FBS and 1% dimethyl sulfoxide (Sigma-Aldrich, St. Louis, MO, USA) on bacteriologic dishes in a humidified incubator containing 5% CO_2_ at 37°C. The medium was replaced every 2 days thereafter. On day 4, the cell aggregates were transferred to cell culture flasks, and on day 10 of differentiation, cells were harvested. The levels of marker proteins of cardiomyocytes, such as cTnT, GATA4 and desmin were detected by western blot assay.

### Quantitative real-time polymerase chain reaction (qRT-PCR)

Total RNA samples were extracted from cultured cells using Trizol reagent (Invitrogen, Carlsbad, CA, USA) according to the manufacturer's protocol, and reverse transcribed into cDNA by using the AMV reverse transcriptase kit (Promega A3500; Madison, WI, USA). miR-20a expression was determined by TaqMan miRNA assays (Ambion, Foster City, CA, USA), and U6 (Applied Biosystems, Foster City, CA, USA) was used as an internal control. The expression of SMO mRNA was determined by PrimeScript RT-PCR kits (Takara Biochemicals, Kyoto, Japan), and β-actin was used as an internal control.

### Western blot assay

Anti-cTnT, anti-β-actin and anti-Ki67 antibodies were purchased from Santa Cruz Biotechnology (Santa Cruz, CA, USA). Anti-GATA4 and anti-desmin were bought from Cell Signaling Technology (Danvers, MA, USA). Anti-SMO, anti-Bax and anti-Bcl-2 antibodies were purchased from Abcam (Cambridge, MA, USA). Cells were added into tubes containing lysis buffer (50 mmol/l Tris-HCl, 0.2% sodium deoxycholate, 0.2% SDS, 1% Triton X-100, and 1 mmol/l EDTA at pH 7.4). The lysate supernatant was collected after centrifugation at 14,000 ***g*** for 30 min at 4°C. Subsequently, protein concentrations were determined using a BCA protein detection kit (Keygen Biotech. Co. Ltd., Nanjing, China) according to the manufacturer's instructions. Western blot assay was performed as previously described (Shen et al., 2012).

### Cell transfection

miR-20a mimics, miR-20a inhibitors, small-interfering RNAs (siRNA) targeting SMO (si-SMO) and their respective controls were obtained from GenePharma (Shanghai GenePharma Co. Ltd., China). P19 cells in exponential growth were plated at a density of 3×10^5^ cells/plate and incubated for 24 h, and then transfected with miR-20a mimics, miR-control, si-SMO or si-control at a 100 nM concentration by using Lipofectamine-2000 (Invitrogen) according to the manufacturer's instructions.

### CCK-8 assay

Cells with 3×10^4^ cells per well were seeded in 96-well plates and cultured in α-MEM containing 10% FBS for 24 h until they were adherent. Cell growth was monitored every day for a period of 5 days, and the proliferation rate was assessed by cell counting Kit-8 (CCK-8; Peninsula Labs, Belmont, CA, USA). Briefly, 5 µl of CCK-8 solution was added to each well, and the plates were further incubated for 2 h. Then the absorbance at 450 and 650 nm was measured by using an ELISA reader, and the differences between the absorbance values were recorded as the optical density.

### Flow cytometry analysis

Cells were harvested using trypsin/EDTA, washed in PBS, resuspended in 1×binding buffer at a concentration of 1×10^6^ cells/ml, and stained with 5 µl annexin V-FITC and 5 µl PI at room temperature for 15 min (BD Pharmingen, San Diego, CA, USA). The FITC and PI fluorescent signals were measured by flow cytometry (BD Biosciences, San Jose, CA, USA).

### Target prediction analysis

Bioinformatics-based target prediction analysis was performed using available bioinformatics logarithms on-line TargetScan 7.0 (http://www.targetscan.org/), PicTar (http://pictar.bio.nyu.edu), and miRBase (http://microrna.sanger.ac.uk/).

### Luciferase reporter assay

The fragment of wild-type SMO 3′-UTR (3′-UTR-WT) containing putative miR-20a binding sites was amplified by PCR, and mutant SMO 3′-UTR (3′-UTR-MUT) was generated by mutating the conserved miR-20a binding sites using an overlap-extension PCR method. The fragment containing 3′-UTR-WT or 3′-UTR-MUT regions of SMO was inserted into psiCHECK-2 vectors (GenePharma, Shanghai, China) containing both renilla and firefly luciferase reporter genes. Subsequently, the psiCHECK-2 vectors which contained wild-type or mutant 3′-UTR sequences of SMO were transfected into miR-20a-overexpressing cells. After 24 h, the luciferase activities of firefly and renilla were assessed using dual luciferase reporter assay system (Promega) in accordance with the manufacturer's protocol.

### Statistical analysis

The GraphPad Prism 5 software (GraphPad Software, San Diego, CA, USA; http://www.graphpad.com) was used for all statistical analyses. Data were expressed as mean±standard error (s.e.m.) or standard deviation (s.d.). The difference between groups was examined by Student's *t*-test. Values of *P<0.05, **P<0.01 and ***P<0.001 were considered statistically signiﬁcant.
